# A PubMed search filter for efficiently retrieving exercise training studies

**DOI:** 10.1186/s12874-024-02414-z

**Published:** 2024-12-18

**Authors:** Dawei Yin, Mikaela V. Engracia, Matthew K. Edema, David C. Clarke

**Affiliations:** Department of Biomedical Physiology and Kinesiology, 8888 University Dr., Burnaby, BC V5A 1S6 Canada

**Keywords:** Search filter, Search hedge, Exercise training, Kinesiology, PubMed, Evidence-based practice, Information storage and retrieval

## Abstract

**Background:**

A barrier to evidence-informed exercise programming is locating studies of exercise training programs. The purpose of this study was to create a search filter for studies of exercise training programs for the PubMed electronic bibliographic database.

**Methods:**

Candidate search terms were identified from three sources: exercise-relevant MeSH terms and their corresponding Entry terms, word frequency analysis of articles in a gold-standard reference set curated from systematic reviews focused on exercise training, and retrospective searching of articles retrieved in the search filter development and testing steps. These terms were assembled into an exercise training search filter, and its performance was assessed against a basic search string applied to six case studies. Search string performance was measured as sensitivity (relative recall), precision, and number needed to read (NNR). We aimed to achieve relative recall ≥ 85%, and a NNR ≥ 2.

**Results:**

The reference set consisted of 71 articles drawn from six systematic reviews. Sixty-one candidate search terms were evaluated for inclusion, 21 of which were included in the finalized exercise-training search filter. The relative recall of the search filter was 96% for the reference set and the precision mean ± SD was 54 ± 16% across the case studies, with the corresponding NNR = ~ 2. The exercise training search filter consistently outperformed the basic search string.

**Conclusion:**

The exercise training search filter fosters more efficient searches for studies of exercise training programs in the PubMed electronic bibliographic database. This search string may therefore support evidence-informed practice in exercise programming.

**Supplementary Information:**

The online version contains supplementary material available at 10.1186/s12874-024-02414-z.

## Background

Exercise and active rehabilitation professionals, such as kinesiologists, physiotherapists, strength and conditioning coaches, exercise physiologists, and personal trainers, design and administer personalized exercise training programs to help their clients achieve health, rehabilitation, and physical performance goals. These professionals are increasingly encouraged to apply evidence-based practice to inform their programming decisions. Evidence-based practice is the notion that clinical decisions should be based on the best current scientific evidence while acknowledging individual patient needs allowing for informed decision-making and transfer into clinical practice [1, 2]. A key task in evidence-based practice is systematically searching the scientific literature. Typically, searches are performed using electronic bibliographic databases like PubMed and SPORTDiscus. However, the quantity of research studies in exercise science makes the search process time-consuming. A further challenge is distinguishing studies of *exercise training*, which feature repeated bouts of exercise over time to build fitness, from studies featuring *acute exercise*, i.e., a single bout of exercise to study physiological responses. Searches for exercise training studies commonly retrieve both exercise training and acute exercise studies, which reduces the specificity of the search and requires the searcher to sift through more articles. These challenges present a barrier to evidence-based practice.

Electronic bibliographic databases feature sophisticated search tools. One such tool is the *controlled vocabulary*, which is a standard set of terms that are used to express the main topics of an article. PubMed’s controlled vocabulary is called Medical Subject Headings or MeSH terms. A MeSH term that reflects the concept of exercise training is “Physical Conditioning, Human” [3]. This MeSH term, introduced in 2014, is defined as “diet modification and physical exercise to improve the ability to carry out daily tasks and perform physical activities” [3]. This MeSH term can thus be used to retrieve studies that employ exercise training to elicit fitness- and performance-enhancing adaptations in humans. It increases the efficiency of searches by excluding studies featuring animal models, studies of acute physiological adaptations to exercise, and studies focusing on tangential aspects of exercise training such as warm-up and cool-down exercises.

While useful, MeSH terms feature the following limitations. First, new MeSH terms can only be applied to articles published after the term was introduced. In the case of “Physical Conditioning, Human,” only articles published in 2014 and thereafter stand to be coded using this term. Second, it takes time for the article coders to read and code new articles, such that newly published articles will take several months to be assigned MeSH terms. Therefore, relatively few exercise training studies are assigned the “Physical Conditioning, Human” MeSH term. Exercise and rehabilitation professionals would benefit from a search tool that could retrieve studies of exercise-training programs published at any time.

One way to address this challenge is to create a *search filter* for exercise training programs. A search filter is a pre-written search string for retrieving studies on a specific topic. Typically, search filters have been formally evaluated for their retrieval properties, such as sensitivity and precision. To our knowledge, no search filters exist for exercise training. However, a few search filters exist that pertain to studies of physical activity. One such filter is designed primarily to search literature from Brazilian academic institutions [4]. Another filter constitutes a complex exercise, PA, play, and sports search hedge for use in the Ovid MEDLINE database [5]. Both filters are unsuitable for finding exercise training studies.

The purpose of this study was to develop and evaluate a search filter for efficiently retrieving studies of exercise-training programs in PubMed. The PubMed database is advantageous for several reasons: it indexes studies within the medical and life science disciplines, it currently indexes over 34 million citations, and it is freely available via the Internet for anyone to use [6]. The search string was evaluated in two ways. First, its sensitivity and precision were estimated by the number of articles retrieved from a gold-standard reference set of articles. Second, its performance was evaluated against a basic search string in six case studies that pertained to scenarios faced by different exercise and rehabilitation professionals. Implementation of the search filter by these professionals therefore promises to assist them in applying evidence-based practice by enabling more effective and time-efficient evidence searching. In turn, applying evidence-based practice will help ensure that their clients and patients are prescribed exercise training that is maximally safe, effective, and time efficient, as defined by the latest scientific research.

## Methods

**Fig. 1:**
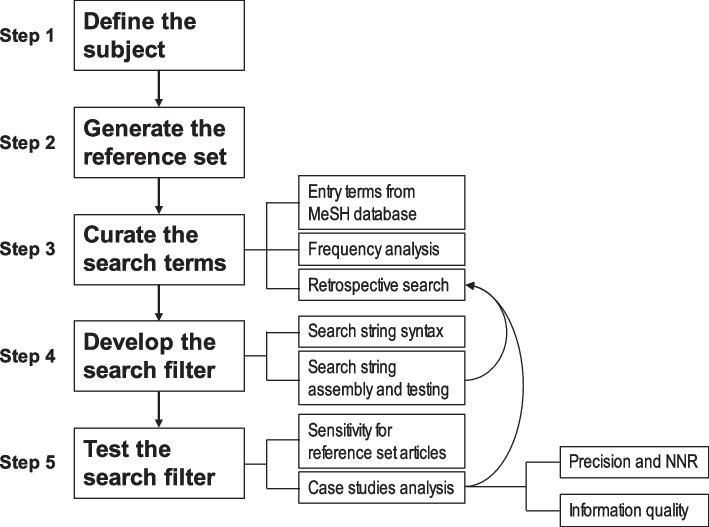
Search filter development steps

The study design featured five phases (Fig. 1), which were specified based on a previously published review on search filter development and the principles of evidence-based practice (EBP) [7, 8].

### Define the subject

We sought to develop a search filter for the “Intervention” concept of the Person-Intervention-Comparison-Outcome (PICO) question format, wherein the intervention is exercise training. The nature of the exercise training programs was deliberately kept broad, such that the resulting search strings could broadly generalize to diverse persons and outcomes, such as those seeking to improve their health, rehabilitate from injuries, or improve their physical performance.

We define *exercise training programs* as a structured plan consisting of physical exercises or activities and their corresponding volumes and intensities to achieve health, fitness, rehabilitation, or performance goals. Articles with exercise training as the main intervention were therefore deemed relevant. To reflect the fact that training programs must be applied for a sufficient duration for adaptations to manifest, we specified that the programs must be implemented for a minimum of two weeks. Indeed, both endurance and resistance training programs can result in observable and relatively stable adaptations within a few weeks [9]. We also sought for the search filter to be inclusive with respect to the types of exercise training modalities featured in the studies. Typically, exercise training programs feature modalities such as resistance, endurance, interval, and circuit-based training applied either alone or in combination. The volume and intensity of training are commonly expressed through the FITT principle, which stands for Frequency, Intensity, Time, and Type. Additional training modalities such as core training and sprint training may also be of interest. Accordingly, a search filter able to retrieve studies of exercise programs with diverse terminology was desired.

### Generate the reference set

To evaluate the sensitivity of candidate search filters, we created a “gold standard” reference set of articles by leveraging existing systematic reviews (SR) that contain high-quality relevant articles [10]. First, we inputted the following search string into PubMed to retrieve systematic reviews regarding exercise training programs: "physical conditioning, human"[mh] OR "exercise therapy"[mh:noexp] OR "aquatic therapy"[mh:noexp] OR (exercise[tw] AND (training[tiab] OR "program*"[tiab])) AND (strength OR endurance) NOT "animals"[mh:noexp]. We call this string the “systematic review search string.” This string features three concepts: intervention (exercise training), outcomes (strength and endurance), and exclusion (animals). Due to the vast number of SRs related to exercise training on PubMed, we limited to only using the SRs published in 2018. We chose 2018 because the reviews would have had sufficient time to have been assigned MeSH terms by National Library of Medicine indexers by the beginning of this study in mid-2022.

We screened the SRs for inclusion as follows. First, the included records had to involve an exercise training program as a primary intervention. SRs that did not involve exercise programming as an intervention or that were combined with topics of diet and nutrition, supplements, or pharmaceuticals were excluded. Furthermore, SRs that lacked abstracts, links to full texts, or were written in a language other than English were also not included. From the pool of included studies, six systematic reviews were randomly selected to include a range of different topics to allow for generalizability when using the filter. The studies included in the final six systematic reviews were extracted and screened to ensure the intervention durations were longer than two weeks.

### Curate the search terms

With the reference set of articles in hand, we curated candidate search terms in the following three ways:

#### Entry terms from the MeSH database

MeSH terms related to exercise programming were obtained by inspecting the reference set and by examining the PubMed MeSH database (e.g., exercise therapy, aquatic therapy, etc.). The Entry terms listed for each MeSH term were then examined for inclusion as keywords in the search filter. Entry Terms are synonyms or related alternate forms of the MeSH terms [11]. Entry Terms were selected as keywords if they were commonly used in the study titles or abstracts from the reference set; Entry Terms that failed to retrieve articles from the reference set were not included.

#### Frequency analysis

We derived additional keywords by applying *word frequency analysis* to each article’s title, abstract, and keywords. Word frequency analysis identifies words or phrases that are commonly used to represent themes and concepts. We performed the frequency analysis using the freely available text miner/word frequency counter WriteWords [12]. Single and double-word phrases were analyzed to identify exercise-training-related terms that recurred most frequently.

#### Retrospective search

Retrospective searching was performed after by iteratively adding candidate search terms appearing in papers retrieved by candidate search strings and searches performed for the case studies. In this case, we extracted candidate keywords from relevant articles retrieved by the basic search string during the search filter testing of the reference set and case studies analysis (described below in Sect. 2.5).

### Develop the search filter

Candidate keywords and MeSH terms were first individually tested for their sensitivity, precision, and NNR with respect to the reference set. Some search terms were modified to maximize retrieval of relevant studies by employing PubMed characters, syntax, and codes such as quotation marks, asterisks, and field tags. The best performing terms were then sequentially assembled using Boolean Operators AND and OR as appropriate, and the candidate search strings were tested for their search properties. Terms found from the retrospective search of the reference set and case study analysis were evaluated in the same manner and were either included or removed from the final filter depending on whether they improved its performance.

#### Search filter syntax

Specific terms and phrases were enclosed in double quotation marks, while the wildcard symbol (*) was appended to the root portion of words to search for keywords that varied in spelling, plural forms, or conjugations of words. For example, the search term ‘program*’ would retrieve articles containing the words programs, programmes, and programming. Quotations marks and asterisks were combined for the search of an exact phrase and with varying suffixes such as “training program*”. This format was made available in PubMed according to its user guide last updated in 2022 [13]. The search string was constructed through an iterative process by manually testing terms within and across concept groups using the Boolean Operators AND and OR as appropriate. In the case studies analysis described below, we added a string component for exclusions using the NOT operator. This approach reduced the number of nonrelevant articles in cases in which excessive nonrelevant articles were retrieved by the “core” search strings (defined below).

Search field tags were also tested and combined with each term to maximize the search string performance. The PubMed search engine searches multiple fields for each indexed article, e.g., abstract, titles, Mesh terms, authors, journal names, etc. [14]. In some cases, searching all fields will reduce the precision of searches. Therefore, the “[tiab]” field tag was employed to search for terms only within the title and abstract, which limited the retrieval of articles featuring the term in those fields and not in others.

#### Assembling and evaluating the candidate search strings

Candidate search strings were evaluated based on the performance metrics of sensitivity, precision, and NNR. *Sensitivity* is constitutively defined as the proportion of relevant articles retrieved from all articles pertaining to a topic [7]. Operationally, we evaluated the sensitivity of our candidate search strings using the *relative recall* metric, which is the proportion of records retrieved from all *retrievable* relevant records [10]. Relative recall is a more practical approach to sensitivity that uses systematic reviews to define a gold-standard reference set, rather than performing the systematic search process oneself. In our case, relative recall was calculated as the number of papers retrieved divided by the total number of papers in the reference set. To restrict the searches to the reference set, all candidate strings were joined with the AND operator to a string containing the PubMed Identifier (PMID) numbers for each article in the reference set linked by the OR operator. The search results would therefore only include the articles from the reference set that were retrieved by the candidate string, thus enabling the straightforward estimation of relative recall.

We also evaluated precision, which is the number of relevant articles amongst the retrieved citations, and its inverse, the NNR, which is the number of studies that need to be read to find a study of sufficient quality and relevance [7, 15]. We aimed for the final search filter to achieve ≥ 85% for the sensitivity and an NNR of 2.2. During the assembly of the search filter, we evaluated precision of candidate strings in an ad hoc manner by documenting the total number of hits retrieved by the strings. Candidate strings that achieved high relative recalls with a reasonable number of articles retrieved were retained.

We assembled the candidate search strings as follows. First, we measured the sensitivities of each MeSH term and of the most frequently appearing keywords from the word frequency analysis. These terms were then sequentially added to the string, and the string was then tested for relative recall. Terms discovered through retrospective searching of articles retrieved during the case study analysis were added to the latest candidate search string, which was then assessed for relative recall and precision. Terms that improved the string’s performance were retained in an updated version of the search string. We refer to the final search string resulting from this process as the *exercise training search filter*.

### Testing the search filter

#### Relative recall of the exercise training search filter

To evaluate the sensitivity of the exercise training search filter, we compared the relative recall of the filter against a basic search string for the reference set. The basic search string was “*exercise[mh] OR exercise[tw]”*. The MeSH term “Exercise” was introduced in 1989 and is categorized under the headings “Motor Activity” and “Human Activities” in the MeSH tree. Eleven main “branches” extend from the exercise concept, which pertains to more specific types or facets of exercise [16]. The word “exercise” is used in everyday vocabulary, such that it was included as a keyword. The [tw] tag refers to “text words”, meaning the term “exercise” could appear in the article’s title, abstract, or one of several other fields (see https://pubmed.ncbi.nlm.nih.gov/help/#tw for details).

#### Search filter performance in case studies

We evaluated the exercise training search filter for its precision, sensitivity, and ability to retrieve strong evidence for supporting exercise programming decisions in six case studies. The case studies were specified to reflect a broad range of realistic patient cohorts and goals that exercise and rehabilitation professionals encounter in their daily practices. Specifically, they feature persons of varying ages and sexes, some of whom are workers or athletes, and some of whom have noncommunicable chronic diseases or musculoskeletal injuries. The goals relate to improving health, rehabilitation, and performance. The six case studies were as follows:

1. A sedentary adult male with type 2 diabetes seeking to improve cardiovascular health.
2. A youth male soccer player seeking to improve sprint speed.
3. An elderly woman with arthritis in most of her joints seeking to reduce pain and improve strength and balance.
4. An adult male construction worker who fell and sustained a low back injury seeking to reduce pain and improve strength.
5. A female adolescent basketball player who sustained an ACL tear seeking to regain strength post-surgery.
6. An adult woman who was in a car accident and sustained whiplash seeking to improve neck mobility and regain strength.

For each case, the basic search string and exercise training search filter were each deployed as the “I” concept in the PICO framework within the three following search strings: the “core” search string featuring the P, I, and O search concepts (C), the “core + NOT” search string that added excluded terms to the core strings (CE), and “core + exclusions + filtered” search (CEF) in which the results of the “CE” strings were filtered for the publication types of systematic reviews, meta-analysis, narrative reviews, and randomized controlled trials (RCT). These publication types are considered highest in the hierarchy of evidence.

The total hits and number of relevant articles were documented for each string. The precision and NNR were then calculated as per the definitions stated above. For an article to be deemed relevant, it had to include an exercise training intervention that lasted at least two weeks and the study had to feature the population and outcomes specified in the case study. This information was collected from the titles and abstracts of the resulting articles. The percentage of relevant articles in the first 150 articles retrieved served as an index of sensitivity.

To appraise the information quality from the retrieved articles, the exercise programming information (FITT principle, exercise contraindications) from the first two to six relevant publications resulting from the CE strings featuring the exercise training search filter was summarized and compared against a gold standard reference. For cases 1, 3, and 4, which featured clinical cases involving individuals with disease or disability, the American College of Sports Medicine’s (ACSM) *Resources for the Exercise Physiologist: A Practical Guide for the Health Fitness Professionals* was used. For cases 2 and 6, systematic reviews were used as the gold standard, because they are considered the highest level of evidence and provide an unbiased consensus from multiple studies on a specific topic. For case 5, the Melbourne ACL Rehabilitation Guide 2.0 was used as the gold standard reference [17].

## Results

### Identification of the gold-standard reference set of articles

**Fig. 2:**
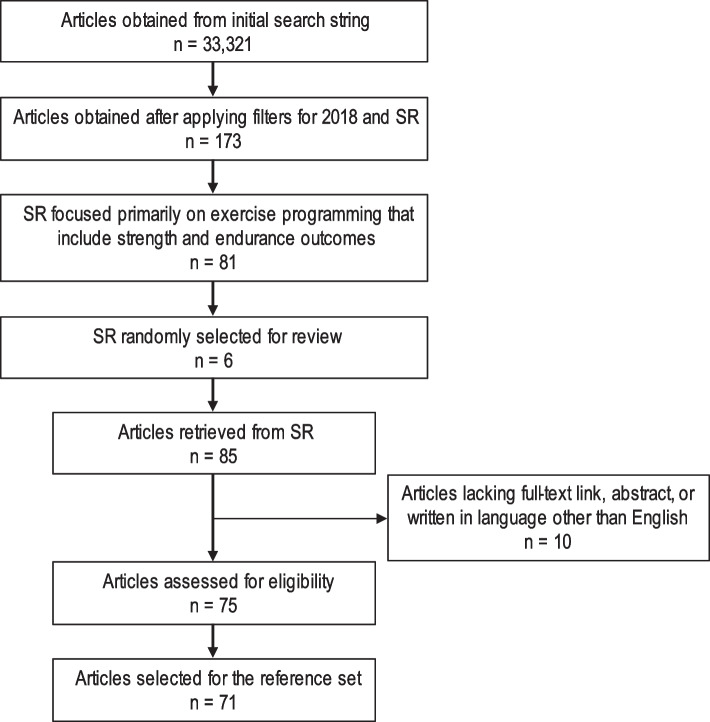
Flow diagram of the screening and identification process of the reference set. SR denotes systematic reviews

The systematic reviews search string retrieved 173 articles after filtering by year (2018), publication type (Systematic Review), language (English), and linked full text. Of the 173 articles, 81 were considered eligible, and six articles were randomly selected from the 81 [18–23]. From these six systematic reviews, 85 RCTs were obtained, of which 71 met the inclusion criteria for the reference set (Fig. 2). The articles reported exercise training studies featuring diverse population cohorts, e.g., athletes, healthy individuals from various age groups, and patient populations (Additional file 1).

### Extraction of search terms

Ten MeSH terms and six Entry Terms were extracted from searching the MeSH database (Table 1). Word frequency analysis of the titles, abstracts, and keywords of the reference set articles produced ten candidate keywords (Table 1). Thirty-five keywords were identified by retrospective searching.

**Table 1:** Candidate search terms resulting from the search term extraction step

Candidate MeSH terms [mh] and keywords from Entry terms	Candidate keywords from frequency analysis	Candidate keywords from retrospective searching	
Physical Conditioning, Human [mh] Exercise Therapy [mh] Resistance Training [mh] Endurance Training [mh] High-Intensity Interval Training [mh] Circuit-Based Exercise [mh] Plyometric Exercise [mh] Physical Education and Training [mh] Exercise Movement Techniques [mh] Aquatic Therapy [mh] physical conditioning resistance training endurance training resistance program* strength program* circuit training	exercise training exercise program* training program* strength training aerobic training interval training balance training sprint interval concurrent training aquatic training	exercise intervention exercise-based intervention game-based physical activity activity program activation training rebound exercise conditioning training plyometric exercise running training football training soccer training fitness training human physical conditioning team training small-sided games physical exercise physical training flexibility exercise stability exercise	muscle training postsurgery rehabilitation prehabilitation program neuromuscular training prevention program rehabilitation program exercise therapy active rehabilitation therapy program active intervention swim training training method resistance exercise weight lifting aquatic exercise program*

“Training” (404) and “Exercise” (174) were the most frequent single words (Table 2). The most prevalent double-word phrases were terms containing “training” (Table 2). The phrases with the highest frequencies were ‘Interval training’ and ‘Resistance training’ with 48 and 39 occurrences, respectively.

**Table 2:** The frequencies of words and phrases retrieved from the frequency analysis

Words	Frequency	Phrases	Frequency
Training	404	Interval training	48
Exercise	174	Resistance training	39
Strength	167	Exercise program	24
Intensity	116	Training program	22
Muscle	113	Concurrent training	20
Physical	90	Strength training	19
Aerobic	82	Aerobic training	15
Performance	76	Training period	15
Interval	75	Endurance training	14
Sprint	70	Sprint interval	12
Resistance	68	Exercise programme	12
Program	66	Exercise training	11
Intervention	62	Sprint training	9
Fitness	56	Weight lifting	8
		Progressive resistance	6
		Volume training	5
		Training programme	5
		Exercise intervention	5
		Based training	5
		Balance training	5
		Aquatic training	3

### Search string development: syntax

The MeSH terms were tested for their relative recall when applied to the reference set. “Exercise[mh]” retrieved the most articles, 52 of the 71 (73.2%) articles in the reference set, but it retrieved a total of 236,835 articles, indicating poor precision. We noticed that using single terms alone in searches substantially reduced the precision of the search; we therefore combined terms using the AND operator or quotation marks, along with different search syntax to find the terms with the highest sensitivities and precision resulting in the best-performing search filter. We also reasoned that the lack of precision could be caused by the automatic explosion of the MeSH terms, in which more specific MeSH terms lower in the hierarchy are also included in the search. We therefore specified “no explosion” for most MeSH terms by appending the search tag syntax *[mh:noexp]*. We did not restrict explosion for the “Physical Conditioning, Human” term, because doing so enabled the search to retrieve articles all facets of exercise training listed below the “Physical Conditioning, Human” term (e.g., “resistance training”[mh], “plyometric exercise”[mh], “endurance training”[mh], etc.) without these MeSH terms needing to be explicitly included in the string.

We added the *[tiab]* search tag to the keywords and phrases. We also tried expressing other phrases as single terms joined with the AND operator (e.g., training[tiab] AND program*[tiab]). While doing so yielded the highest relative recall values, it substantially lowered the precision because any article featuring both terms somewhere in the abstract or title was retrieved. In general, phrases were enclosed in double quotes to force a phrase search, which improved the precision. There were a few exceptions to this strategy. For example, we included the term Exercise[mh:noexp] AND training[tiab], because it allows for the retrieval of articles containing “training” and the MeSH term “exercise” without its subsequent branches in the tree due to automatic explosion. The finalized search terms used in our exercise training search filter are listed in Table 3.

**Table 3:** Finalized terms in the exercise training search filter

MeSH terms [mh] and Entry terms	Keywords retrieved from frequency analysis	Keywords retrieved from retrospective searching
Physical Conditioning, Human [mh] Exercise Therapy [mh:noexp] Aquatic Therapy [mh] Exercise Movement Techniques [mh:noexp] Exercise [mh:noexp] Resistance training Endurance training	Training Exercise* Exercise training Interval training Training program* Exercise program* Concurrent training Aerobic training Sprint interval Aquatic training	Fitness training Swim training Game-based Weight lifting

### Search filter development: assembly and testing

Evaluation of strings that included terms found from the MeSH database, the Entry terms, and the word frequency analysis yielded the following string that had high relative recall and good precision: "Physical Conditioning, human"[mh] OR "Exercise therapy"[mh:noexp] OR "Aquatic therapy"[mh] OR "Exercise Movement Techniques"[mh:noexp] OR "Interval training"[tiab] OR "Training program*"[tiab] OR "Exercise program*"[tiab] OR "Resistance training"[tiab] OR "Concurrent training"[tiab] OR "Aerobic training"[tiab] OR "Endurance training"[tiab] OR "Sprint interval"[tiab] OR "Aquatic training"[tiab] OR "Exercise training"[tiab] OR (Exercise[mh:noexp] AND training[tiab]).

In the search filter testing, the basic search string retrieved articles that were retrospectively searched for potential keywords that were not present in the articles retrieved using the candidate string above, i.e., the initial “comprehensive string”. Four terms improved relative recall while maintaining or improving precision, and these were appended to the string as follows: OR "fitness training"[tiab] OR ("weight lifting"[tiab] AND exercise*[tiab]) OR "swim training"[tiab] OR ("game-based"[tiab] AND training[tiab]). The same retrospective search process was applied to the articles found from each case study analysis, which resulted in 32 additional candidate keywords (e.g., physical activity, neuromuscular training, rehabilitation program, etc.; Table 1). However, none were included in the final search filter. We listed them in Table 1 for readers who may wish to use them in customized strings based on their needs. The finalized exercise training search filter was therefore as follows: "Physical Conditioning, human"[mh] OR "Exercise therapy"[mh:noexp] OR "Aquatic therapy"[mh] OR "Exercise Movement Techniques"[mh:noexp] OR "Interval training"[tiab] OR "Training program*"[tiab] OR "Exercise program*"[tiab] OR "Resistance training"[tiab] OR "Concurrent training"[tiab] OR "Aerobic training"[tiab] OR "Endurance training"[tiab] OR "Sprint interval"[tiab] OR "Aquatic training"[tiab] OR "Exercise training"[tiab] OR (Exercise[mh:noexp] AND training[tiab***])*** OR "fitness training"[tiab] OR ("weight lifting"[tiab] AND exercise*[tiab]) OR "swim training"[tiab] OR ("game-based"[tiab] AND training[tiab]).

### Search filter testing

#### Sensitivity: comparison against the basic search string

The exercise training search filter achieved higher relative recall compared to the basic search string while retrieving 69% fewer articles than the basic search string (Table 4).

**Table 4:** Properties of the exercise training search filter

Search string	Performance		
	Retrieved (out of *N* = 71)	Relative recall (%)	Total articles retrieved
Basic search string	64	90.1	488,901
Exercise training search filter	68	95.8	151,835

#### Precision and NNR: case studies analysis

The exercise training search filter retrieved more relevant articles and fewer total articles than the basic search string, resulting in higher precision and lower NNR (Table 5). Across the six case studies and three strings, the exercise training search filter increased precision by an average of 1.5-fold compared to the basic search string, which corresponded to an average reduction in the NNR from approximately three to two papers. Broken down by the full search strings, the exercise training search filter increased precision by 1.4-, 1.9-, and 1.2-fold for the C, CE, and CEF search strings, respectively. The most pronounced difference was in case 1, for which the exercise training search filter exhibited 1.6-, 3.5-, and 1.7-fold increases in precision compared to the basic search string for the C, CE, and CEF strings, respectively. In contrast, equivocal results were obtained in case 5, for which the basic search string exhibited higher precision for the C and CEF strings (50% vs. 40% and 56% vs. 55%, respectively), whereas the exercise training search filter had higher precision for the CE string (40% vs. 25%).

**Table 5:** Case studies analysis

Component strings for the P and O concepts and excluded terms (E)		Full search strings^a^	Intervention component string	Performance					
				Retrieved	Relevant^b^	Precision (%)	NNR	Precision fold	NNR fold
Case #1	P: (Adult[mh] OR adult^*^) AND (Male[mh] OR Men[mh] OR male^*^) AND ("Diabetes Mellitus, Type 2"[majr] OR "type 2 diabetes") O: ("vascular stiffness"[tiab] OR "vascular health"[tiab] OR "arterial stiffness"[tiab] OR "vascular function"[tiab] OR "cardiovascular function"[tiab] OR "cardiac function"[tiab] OR "cardiovascular health"[tiab] OR "cardiovascular risk"[tiab] OR "blood pressure"[tiab] OR “blood pressure”[mh]) E: NOT ("type 1 diabetes") NOT (nutrition therapy[mh]) NOT (elderly[tiab] OR "older adult^*^"[tiab] OR "older patient^*^"[tiab] OR "older people"[tiab] OR Aged[mh]) NOT ("diet therapy"[mh] OR "diet"[tiab])	C	Basic	402	45	30.0	3.3		
			Filter	174	87	58.0	1.7	1.9	0.52
		CE	Basic	348	27	18.0	5.6		
			Filter	81	51	63.0	1.6	3.5	0.29
		CEF	Basic	87	35	40.2	3.3		
			Filter	46	31	67.4	1.5	1.7	0.45
Case #2	P: (“Soccer”[mh] OR “Soccer”[tiab]) AND ("Adolescent"[Mesh] OR adolescen^*^ OR teen^*^ OR youth OR juvenile^*^) AND (Male[mh] OR Men[mh] OR male^*^) O: (sprint^*^ OR "sprint ability" OR "sprint speed" OR “Athletic Performance”[mh]) E: NOT (nutrition therapy[mh]) NOT (diseases category[mh]) NOT (wounds and injuries[mh]) NOT ("cancer") NOT (basketball OR "ice hockey" OR football)	C	Basic	726	45	30.0	3.3		
			Filter	332	72	48.0	2.1	1.60	0.64
		CE	Basic	370	45	30.0	3.3		
			Filter	183	81	54.0	1.9	1.80	0.58
		CEF	Basic	98	57	58.7	1.7		
			Filter	69	52	75.4	1.3	1.28	0.76
Case #3	P: (elderly[tiab] OR "older adult^*^"[tiab] OR "older patient^*^"[tiab] OR "older people"[tiab] OR Aged[mh] OR “Seniors”[tiab]) AND (Female[mh] OR Women[mh] OR female^*^) AND ("Osteoarthritis"[mh] OR “Osteoarthritis”[tiab]) O: ("Muscle Strength"[mh] OR "Strength^*^"[tiab] OR "Pain Management"[mh] OR “pain”[tiab] OR “Reduce Pain"[tiab] OR "Postural Balance"[mh] OR “Balance”[tiab]) E: NOT (nutrition therapy[mh]) NOT ("diet therapy"[mh] OR "diet"[tiab]) NOT ("Adolescent"[Mesh] OR adolescen^*^ OR teen^*^ OR youth OR juvenile^*^) NOT ("Musculoskeletal Manipulations"[mh]) NOT ("Acute Pain"[mh]) NOT ("Arthroplasty"[mh]) NOT (Tape[tiab] OR Taping[tiab]) NOT ("Laser Therapy"[mh]) NOT ("Ultrasonic Therapy"[mh] OR "Ultrasound"[tiab]) NOT ("Electric Stimulation Therapy"[mh]) NOT ("Cryotherapy"[mh]) NOT ("Hyperthermia, Induced"[mh])	C	Basic	1,656	47	31.3	3.2		
			Filter	767	73	48.7	2.1	1.56	0.66
		CE	Basic	1,105	48	32.0	3.1		
			Filter	514	90	60.0	1.7	1.88	0.55
		CEF	Basic	442	84	56.0	1.8		
			Filter	311	109	72.7	1.4	1.30	0.78
Case #4	P: (Adult[mh] OR adult^*^) AND (Male[mh] OR Men[mh] OR Male^*^) AND (("Low Back Pain"[mh] OR "Low back pain"[tiab] OR "low back injury"[tiab]) OR ((“Lumbosacral Region”[mh] OR “back muscles”[mh] OR “lower back”) O: ("Range of Motion, Articular"[mh] OR "Range of motion"[tiab] OR “flexibility”[tiab] OR "mobility"[tiab] OR "Muscle Strength"[mh] OR "Strength^*^"[tiab] OR "Pain Management"[mh] OR “pain”[tiab] OR "Return to Work"[mh]))) E: NOT (nutrition therapy[mh]) NOT ("diet therapy"[mh] OR "diet"[tiab]) NOT ("Adolescent"[Mesh] OR adolescen^*^ OR teen^*^ OR youth OR juvenile^*^) NOT ("Dry Needling"[mh]) NOT ("Musculoskeletal Manipulations"[mh]) NOT (Chronic Pain[mh]) NOT (elderly[tiab] OR "older adult^*^"[tiab] OR "older patient^*^"[tiab] OR "older people"[tiab] OR Aged[mh]) NOT ("Sports"[mh])	C	Basic	2,621	46	30.7	3.3		
			Filter	1,320	67	44.7	2.2	1.46	0.67
		CE	Basic	1,056	45	30.0	3.3		
			Filter	572	72	48.0	2.1	1.60	0.64
		CEF	Basic	342	83	55.3	1.8		
			Filter	256	95	63.3	1.6	1.14	0.89
Case #5	P: (Female[mh] OR Women[mh] OR Female^*^) AND ("Adolescent"[mh] OR adolescen^*^ OR teen^*^ OR youth OR juvenile^*^) AND ("Anterior Cruciate Ligament Injuries"[mh] OR "Anterior Cruciate Ligament"[tiab] OR "ACL"[tiab]) O: ("Return to Sport"[mh] OR "Return to Sport"[tiab] OR "Muscle Strength"[mh] OR "Strength^*^"[tiab] OR “Return to Play”[tiab]) E: NOT ("Injury Prevention"[tiab]) NOT (elderly[tiab] OR "older adult^*^"[tiab] OR "older patient^*^"[tiab] OR "older people"[tiab] OR Aged[mh]) NOT (nutrition therapy[mh])	C	Basic	55	27	49.1	2.0		
			Filter	47	19	40.4	2.5	0.82	1.25
		CE	Basic	164	38	25.3	3.9		
			Filter	81	32	39.5	2.5	1.56	0.64
		CEF	Basic	43	24	55.8	1.8		
			Filter	33	18	54.5	1.8	0.98	1.00
Case #6	P: (Adult[mh] OR adult^*^) AND (Female[mh] OR Women[mh] OR female^*^) AND (“Whiplash Injuries”[majr]) AND (“Neck Injuries”[majr]) O: ("Acute Pain"[tiab] OR "Sprains and Strains"[tiab] OR "Neck Muscles"[tiab] OR "Range of Motion, Articular"[tiab] OR "Neck Pain"[tiab] OR "Neck"[tiab]) E: NOT (chemicals and drugs category[mh]) NOT (nutrition therapy[mh]) NOT ("diet therapy"[mh] OR "diet"[tiab]) NOT ("Adolescent"[Mesh] OR adolescen^*^ OR teen^*^ OR youth OR juvenile^*^) NOT (“Dry Needling”[tiab]) NOT (“Musculoskeletal Manipulations”[tiab]) NOT (Chronic Pain[tiab])	C	Basic	70	47	67.1	1.5		
			Filter	43	36	83.7	1.2	1.25	0.80
		CE	Basic	50	36	72.0	1.4		
			Filter	33	27	81.8	1.2	1.14	0.86
		CEF	Basic	25	21	84.0	1.2		
			Filter	21	18	85.7	1.2	1.02	1.00

^a^*C* core search string, *CE* core + exclusions, *CEF* core + exclusions + filtered

^b^For cases in which the total number of articles retrieved by a string exceeded 150, the number of relevant articles was determined only for the first 150 articles. “Precision fold” and “NNR fold” refer to the fold change between the filter and the basic search string for those metrics

#### Quality of information in articles retrieved by the exercise training search filter

For the first case study (type 2 diabetic), the ACSM gold standard recommends aerobic exercise that utilizes large muscle groups in a rhythmic motion performed at an intensity of 50–80% of heart rate reserve (HRR), with duration starting at 20 min and progressing to 60 min per session (Table 6) [24]. The recommended resistance training involves performing exercises at 60–80% 1-repetition maximum (RM) intensity for three sets of ten repetitions [24]. To mitigate the risk of adverse events, the ACSM recommends that high-carbohydrate snacks be available during exercise [24]. Another important consideration is the maintenance of proper foot care because those with diabetes exhibit longer healing times, increasing the risk of infection from foot sores and blisters, potentially resulting in more serious complications [24].

**Table 6:** Case 1 results: FITT guidelines for individuals with type 2 diabetes from a gold standard reference and from information extracted from articles retrieved by the search filter

Exercise mode	Frequency	Intensity	Time	Type
**ACSM**				
Aerobic conditioning	5–7 × /week	50–80% HRR and VO2 reserve RPE = 12–16	Start at 20 min/session and progress to 60 min/session	Any aerobic exercise that utilizes large muscle groups in a rhythmic motion
Resistance exercise	2–3 × /week	60–80% of 1RM 2–3 sets of 8–12 repetitions	-	Free weights, elastic bands, body weight exercises, and machine exercises
**Search filter articles (*N*** **= 2)**				
Aerobic conditioning	3 × /week	50–70% VO2 reserve	Start at 30 min/session and progress to 50 min/session at target HR	Cycle ergometers, treadmills, recumbent steppers, and elliptical trainers
Resistance Exercise	3 × /week	70–80% of 1RM 3 sets of 10 repetitions	-	Chest press, shoulder press, vertical traction, leg press, leg extension, leg curl, abdominal crunch, free weights

The first two relevant articles selected from the results of the exercise training search string yielded similar results (Table 6). These articles recommend performing aerobic conditioning at 50–70% of HRR for 30–50 min involving rhythmical movements that use large muscle groups [25]. For resistance exercise, 70–80% of 1-RM for three sets of ten was recommended [26]. To mitigate the risk of adverse events, the articles recommended that carbohydrate snacks be readily available in the event of hypoglycemia and that foot examinations be performed weekly to inspect for foot ulcers and be treated if necessary to prevent infection [25].

For the second case study (soccer player), three systematic reviews were chosen as the gold standard. These articles recommend plyometrics be performed three to four times per week for more than 80 repetitions total in each session (Table 7) [27, 28]. Recommended exercises include squats, depth jumps, countermovement jumps, agility training, and change of direction exercises, with the jumps being performed both bilaterally and unilaterally [27, 28]. In addition, sprint training is recommended to be performed two to three times per week. Maximal-effort sprints lasting 10–30 s should be performed on a treadmill or outdoors [29]. Twelve sprints should be performed each session with the recovery duration being five times the work duration [29].

**Table 7:** Case 2 results: FITT guidelines for individuals seeking to improve speed from a gold standard reference and from information extracted from articles retrieved by the search filter

Exercise mode	Frequency	Intensity	Time	Type
**Systematic reviews (*N* = 3)**				
Plyometrics	3–4 × /week	> 80 repetitions in total	-	Unilateral and bilateral squats, unilateral and bilateral depth jumps, unilateral and bilateral CMJ’s, lunges, hip thrusts, single leg hip thrust, agility and change of direction exercises
Sprint training	2–3 × /week	12 repetitions Maximal intensity	10–30 s Recovery: 5 × the duration of work	Treadmill or outdoor sprinting
**Search filter articles (*N* = 2)**				
Plyometrics	3 × /week	2 sets of 8 repetitions	-	Unilateral and bilateral box jumps, unilateral and bilateral weighted squat jumps, change of direction exercises
		1 set of 20 repetitions	-	Bilateral footwork drills
		2 sets of 10 repetitions	-	Unilateral footwork drills
		2 sets of 10 repetitions	-	Bilateral foam hops
Sprint training	3 × /week	15–26 repetitions 5–30% grade ≥100% maximal aerobic running speed	6–30 s Recovery: 4 × the duration of work	Treadmill sprinting

The articles from the exercise training search filter prescribed similar exercise training. The articles recommended plyometrics to be performed three times per week, with roughly 80 total repetitions to be executed per session (Table 7) [30, 31]. Example exercises include bilateral and unilateral squats and jumps, footwork agility training, and change of direction exercises [30, 31]. Sprint training was also recommended, with 15–26 repetitions of maximal sprints for three times a week [30]. The prescribed sprint duration was 6–30-s with recovery periods lasting four times the work duration [30].

For the third case study (arthritis), the ACSM gold standard recommends low-impact and lower-intensity aerobic exercise such as walking, cycling, rowing, and swimming for aerobic exercise (Table 8) [24]. For resistance training, they suggest weight machines, isometric exercises, and elastic bands [24]. To train flexibility, they suggest a combination of dynamic and static stretching for all major joints [24]. Exercise intensity should be such that pain is minimized, with the rating of perceived exertion being within 11 to 16 for aerobic exercises and a selected % maximum voluntary contraction that is dependent on pain tolerance for resistance exercises [24].

**Table 8:** Case 3 results: FITT guidelines for individuals with arthritis from a gold standard reference and from information extracted from articles retrieved by the search filter

Exercise mode	Frequency	Intensity	Time	Type
**ACSM**				
Weight-Bearing Aerobic	3–5 × /week	60–80% max HRR RPE = 11–16	Start at 5 min/session and progress to 30 min/session	Walking, cycling, rowing, swimming
Resistance exercise	2–3 × /week	%MVC dependent on pain tolerance	-	Weight machines, isometric exercise, elastic bands
Flexibility/Stretching	Daily	-	30 + seconds per static stretch for 3 reps each side	Dynamic and static stretches for each major joint
**Search filter articles (*N* = 4)**				
Aerobic exercise	3 × /week	40–60% HR max RPE = 13–15	30 min	Walking, stationary cycling
Resistance exercise	2–5 × /week	2–3 sets of 8–10 repetitions Body weight for all weight-bearing exercises (Start at 20% 1RM for leg press and progress by 5–10% increments) RPE = 11–15	-	Seated knee flexion, isometric quad contraction, side lying hip abduction, Narrow and wide stance squat, leg press, step ups, sit to stand, calf raise
Flexibility/Stretching	Daily	3 sets of 10 repetitions	10 s holds	Standing calf stretch, supine hamstring stretch, prone quadricep stretch

The four articles derived from the exercise training search filter results recommended that aerobic, resistance, and flexibility exercises be performed for similar frequencies to those prescribed by the ACSM (Table 8) [32–35]. They likewise recommended similar intensities (RPE ranges) and types of exercises, cautioning on keeping the movements at a lower intensity and free of impact forces and pain [32–35]. For aerobic training specifically, the articles recommended exercises like walking and cycling performed at an RPE of 13–15, which is within the range of 11–16 recommended by the ACSM resource [32–35].

For the fourth case study (low back pain), the ACSM gold standard recommends core exercises such as bridging, bird dog, and curl-ups, to be performed two to three times a week with high reps and low loads, holding each position for six seconds (Table 9) [24]. They also recommend daily stretches such as unloaded spinal flexion and extension stretches, which they state should be held for 30 or more seconds for two to three repetitions [24]. Lastly, they recommend daily aerobic exercises such as fast walking to be performed for 30 min per day [24]. Exercise that increases the intensity or frequency of pain should be avoided [24].

**Table 9:** Case 4 results: FITT guidelines for individuals with low back pain from a gold standard reference and from information extracted from articles retrieved by the search filter

Exercise mode	Frequency	Intensity	Time	Type
**ACSM**				
Weight-bearing Aerobic exercise	Daily	Prescribed % of maximum as tolerated below pain threshold	Aim to achieve 30 min/day	Fast walking
Resistance exercise	2–3 × /week	High reps and low loads	Hold isometric exercises for 6 s	Bridging, bird dog, curl ups
Flexibility and stretching	Daily	Stretch within a pain-free range of motion	Hold for 30 + seconds for 2–3 reps	Limit exercises to unloaded spinal flexion/extension
**Search filter articles (*N* = 4)**				
Core stabilization program	3–5 × /week	5 sets 8–10 repetitions	Hold for 5–10 s	Bird dog, bridging, side plank, prone back extension, and bridging on swiss ball
Flexibility and stretching	5 × /week	-	Hold for 30 s for 3 sets each side	Half-kneeling quad stretch, back flexion/extension mobilizations, erector spinae stretch

The four selected articles proposed similar recommendations. They suggest two of the same core stabilization exercises, bird dog and bridging (Table 9), recommending that each position be held for five to ten seconds [36, 37]. Regarding flexibility and stretching, the articles recommended spinal flexion and extension stretches, holding for 30 s for three sets, the same as that prescribed by the ACSM [38, 39]. With respect to contraindications, the articles advised that pain should be monitored and high-intensity or high-impact exercises should be avoided by individuals with low back pain [38].

For the fifth case study (ACL tear), the Melbourne ACL rehabilitation guide includes prescriptions for strength training, muscular endurance training, balance training, and agility training [17]. The strength training includes exercises such as single-leg bridges, calf raises, single-leg squats, leg presses, and weighted squats (Table 10) [17]. For muscular endurance, the guide mentions the side bridge endurance tests [17]. Balance training involves single-leg standing with eyes open and closed, star excursion balance test, and single-leg stance with head movements [17]. Agility training includes single-leg forward hops, triple hops, triple crossover hops, and side hops [17]. The guide mentions that during early rehabilitation, activities involving impact should be avoided; instead, non-impact activities like cycling, swimming, and walking should be emphasized [17]. It is also important to “listen to the knee”, which they explain as reducing workload in response to pain or swelling [17]. Change of direction training should be gradually reintroduced as well with the supervision of a clinician [17].

**Table 10:** Case 5 results: FITT guidelines for individuals rehabilitating an ACL tear from a gold standard reference and from information extracted from articles retrieved by the search filter

Exercise mode	Frequency	Intensity	Time	Type
**Melbourne ACL rehabilitation guide 2.0**				
Strength training	-	> 20 repetitions and > 85% compared with healthy side	2 s every calf raise repetition	Single leg bridges and calf raises
	-	Phase 2: > 10 repetitions and > 85% compared with healthy side Phase 3: > 22 repetitions	-	Single leg squat
	-	Phase 2: 1RM 1.5 × body weight Phase 3: 1RM 1.8 × body weight	-	Single leg press and squats
Muscular endurance training	-	> 85% compared with the healthy side	Aim to hold for 30 s	Side bridge endurance test
Balance training	-	-	43 s for eyes open 9 s for eyes closed	Unipedal standing (eyes open and closed)
	-	> 95% composite score compared with healthy side	-	Star excursion balance test
	-	At a rate of 60 beats per minute	15 s	Unipedal standing (side to side/up and down head movements)
Agility	- -	> 95% distance compared with healthy side > 95% repetitions compared with healthy side	- 30 s for side hop test	Single hop, triple hop, triple cross over hop Side hops
**Search filter articles (*N* = 3)**				
Strength training	2 × /week	2–3 sets of 5–10 repetitions	-	Nordic hamstrings, standing squats, step-ups, leg raises, single leg leg press, single leg squats
Plyometrics	2 × /week	3 sets of 10 repetitions 10 repetitions of each variation 2–3 sets	- - 10–30 s	Drop jumps Triple single leg hops (forward/backward and side to side) Tuck jumps
Balance	2 × /week	3 sets	30–60 s	Single-leg balance
Agility	2 × /week	3–4 drills per session Start at 50% effort and progress to 100% effort	-	Forward/backward running, side shuffles, cariocas, figure eights, circles, 90 degree turns

The three articles selected from the exercise training search filter proposed exercise training similar to that of the gold standard. Both resources prescribed comparable types of exercises, such as single-leg squats, single-leg balance, triple single-leg hops, and single-leg presses (Table 10) [40–42]. All three articles also mentioned that all exercises should be done under the supervision of a physiotherapist and that the workload will be reduced in response to pain or swelling [40–42]. One study mentioned the importance of learning correct landing techniques before returning to sports [40], in agreement with the Melbourne guide.

For the sixth case study (whiplash), the recommendations from three systematic reviews were summarized. They recommend five to ten repetitions of neck-specific isometric exercises, including chin tucks, neck flexion, extension, and rotation (Table 11) [43, 44]. They also recommend concentric exercises be performed twice per week, involving 20 repetitions of weighted isotonic neck exercises in all cervical planes [43, 44]. To improve neck mobility, they recommend five to ten repetitions of daily active range of motion movements in all cervical planes [43, 44]. Regarding contraindications, those with whiplash-associated disorder individuals may experience exercise-induced hyperalgesia, which is a pressure pain sensitivity that is significantly higher post-exercise [45]. This condition can be prevented by monitoring pain and selecting an intensity that is sufficient for therapeutic benefit but avoids aggravating pain hypersensitivity or symptoms [45].

**Table 11:** Case 6 results: FITT guidelines for individuals with whiplash from a gold standard reference and from information extracted from articles retrieved by the search filter

Exercise mode	Frequency	Intensity	Time	Type
**Systematic reviews (*N* = 3)**				
Neck-specific isometric exercises	Daily	5–10 repetitions	-	Deep neck flexor exercises (chin tuck), isometric neck flexion, extension, and rotation
Resistance neck exercises	2 × /week	20 repetitions	-	Weighted isotonic neck exercises in all cervical planes
Neck mobility exercises	Daily	5–10 repetitions	-	Active range of motion in all cervical planes
**Search filter articles (*N* = 4)**				
Neck-specific isometric exercises	Daily	3 sets 5 repetitions (20 for head lifts)	Hold for 3–5 s	Supine chin tuck, isometric neck flexion, extension, and rotation, prone and supine head lifts
Resistance neck exercises	2 × /week	15–20 repetitions	-	Resisted neck flexion, extension, side flexion, rotation
Neck mobility exercises	Daily	10 repetitions	-	Active range of motion in all cervical planes

The four articles retrieved using the exercise training search filter proposed comparable exercise programming information. These articles recommend similar neck-specific isometric and concentric exercises for similar frequency and number of repetitions [46, 47] (Table 11). For mobility, the articles prescribed ten daily repetitions of active range of motion in all cervical planes [48]. Regarding contraindications, one of the retrieved articles mentioned that sensory hypersensitivity can be induced by aerobic exercise [49]. However, the same study reported that isometric exercise is beneficial for reducing hypersensitivity in those with whiplash-associated disorders [49]. For all six case studies, none of the prescription details retrieved from the articles conflicted with those of the corresponding gold standard resource.

## Discussion

The purpose of this study was to develop a PubMed search filter to efficiently retrieve studies of exercise training programs. We developed the search filter in a five-phase process. Candidate search terms were identified from exercise-relevant MeSH terms, Entry Terms, frequently occurring keywords in a gold-standard reference set of articles, and retrospective searches of articles retrieved during search filter testing. Candidate search strings were evaluated for their relative recall (articles retrieved from the reference set) and the total number of articles retrieved (an index of precision). The final search filter was then compared against a basic search string for its relative recall of articles from the reference set and the total number of articles retrieved and for its precision and NNR when applied to six case studies. Overall, the final filter achieved a relative recall of ≥ 85% for the gold-standard reference set and an NNR of ≤ 2 for all six case studies, outperforming the basic search string in all cases.

To our knowledge, our study is the first to formally develop and evaluate a search filter specific to exercise training studies. The search filter achieved our target goals for both sensitivity and precision and retrieved exercise training information for diverse case studies that agreed with the information from contemporary gold-standard references. The search filter should therefore assist exercise and rehabilitation professionals to find high-quality evidence in a more time-efficient manner than using basic search strings. The results of our study also support the broad generalizability of the exercise training search filter to different exercise programming settings. Should users find that our search filter performs less satisfactorily for their needs, the search filter can be straightforwardly customized by the user. Our study therefore serves as a foundation on which future studies that develop and evaluate improved exercise training search filters can be based.

We recommend that exercise and rehabilitation professionals implement the exercise training search filter as part of a comprehensive evidence-based approach to exercise programming [2]. After completing the intake process for their client or patient, the professional should use the findings to develop the PICO question, then develop the corresponding search strings and conduct the literature search. This part of the process is exemplified in the six case studies conducted in this study. The retrieved articles should be critically appraised, and best evidence pertaining to exercise programming extracted and implemented in designing the client or patient’s program [2]. The professional may need additional information than what is presented in the articles. For example, the ACSM advises the specification of FITT-VP parameters for exercise prescriptions, where V refers to “training volume” and P the rate of progression. In this context, “training volume” refers to the recommended energy expenditure, expressed in units of MET‧min per week or as steps per week if pedometry is used as the measure [50]. For this parameter, general guidelines are available [50], and the Compendium of Physical Activities [51] can be used to estimate the energy expenditures of the prescribed exercises. Similarly, strategies for behavior change, a key consideration of holistic exercise programming [50, 52], may likewise require dedicated evidence searching. Once the exercise program is implemented, the exercise or rehabilitation professional should monitor the training and the outcome variables of interest and adjust the training parameters accordingly to foster continued progress while minimizing the risks of injury or illness.

Our study and the resulting exercise training search filter feature several limitations. First, a tradeoff exists between sensitivity and precision, which we attempted to balance with our search filter. For example, we observed that articles used variations of search terms included in the search filter. The temptation thus existed to add these new terms to the filter, but we observed that doing so was not always beneficial. For example, the three following articles were missed by the search filter: PMID 16181565, 11740194, and 10683055 [53–55]. Adding the terms “physiotherapy” and “resistance exercise” to the search filter enabled them to be retrieved, but at the cost of many additional irrelevant articles being retrieved, thus reducing the precision. In addition, for applications pertaining to sport-specific training, it may be worth combining the sport name with the term “training” followed by the search field tag *[tiab]*. In case study #2, one relevant article was missed but would have been retrieved if the search filter contained the term *“soccer training”[tiab]* [56].

Second, our search filter is specific to the PubMed electronic bibliographic database. While PubMed is widely available and used, it may not be the database of choice for some exercise and rehabilitation professionals, who may prefer databases more specific to their needs such as SPORTDiscus. The relative merits and limitations of databases other than PubMed for retrieving exercise training studies have yet to be systematically studied and represent an avenue for future research. Considering the rapid advance of research and annual changes to PubMed, it will likely be necessary to modify the existing search filter to sustain and improve its functionality.

Third, our approach to generating the gold-standard reference set attempted to balance comprehensiveness with practicality. The 2018 restriction limited the number of studies featured in the gold standard reference set, and with fewer articles than the minimum of 100 recommended by Sampson et al. when using relative recall [10]. Accordingly, the reference set used in our study may not be fully representative of the exercise training literature. Given the observed improvements in search properties conferred by the exercise-training search filter, we propose that this limitation is a minor one.

Finally, the search filter provides no benefit for specifying the portions of search strings for the other search concepts featured in the search. Indeed, users will still require expertise in using PubMed effectively. Adhering to the PICO method provides a framework for organizing the search into discrete concepts. But the user will still need to know useful keywords and corresponding MeSH terms for the P and O concepts. The component search strings from the case study analysis listed in Table 4 can serve as a starting point for interested readers.

## Conclusion

Effective and efficient searches for best evidence require specialized expertise and can be time consuming. To help address these obstacles, we developed and evaluated an exercise training search filter for the PubMed electronic bibliographic database. The search filter demonstrated improved sensitivity, precision, and NNR compared to a basic search string, and is generalizable to diverse exercise programming scenarios. The search filter can be easily incorporated by users as the “I” component of searches that follow the PICO framework and can be straightforwardly modified for customized use. We conclude that the search filter will assist exercise and rehabilitation professionals to employ evidence-based practice.

## Supplementary Information

Supplementary Material 1.

## Data Availability

No datasets were generated or analysed during the current study.
